# Perceived parenting styles and incidence of major depressive disorder: results from a 6985 freshmen cohort study

**DOI:** 10.1186/s12888-023-04712-0

**Published:** 2023-04-05

**Authors:** Jing Cheng, Debiao Liu, Huancheng Zheng, Zhou Jin, Deborah Baofeng Wang, Yan Liu, Yili Wu

**Affiliations:** 1 Cheeloo College of Medicine, Shandong University, Jinan, 250012 China; 2 Center of Evidence-Based Medicine, School of Mental Health, Jining Medical University, Jining, 272013 China; 3 Shandong Key Laboratory of Behavioral Medicine, School of Mental Health, Jining Medical University, Jining, 272013 China; 4 Zhejiang Provincial Clinical Research Center for Mental Disorders, School of Mental Health and The Affiliated Wenzhou Kangning Hospital, Key Laboratory of Alzheimer’s Disease of Zhejiang Province, Oujiang Laboratory (Zhejiang Lab for Regenerative Medicine, Vision and Brain Health), Wenzhou Medical University, Wenzhou, Zhejiang 325000 China; 5 Shandong Collaborative Innovation Centre for Diagnosis, Treatment & Behavioural Interventions of Mental Disorders, Institute of Mental Health, Jining Medical University, Jining, 272013 China

**Keywords:** Parenting styles, Major depressive disorder, Freshmen, Cohort study, Risk factors

## Abstract

**Background:**

Even though a fair amount of studies focus on depression among college students, the effect of perceived parenting styles on the incidence of major depressive disorder (MDD) among representative freshmen in Chinese context is scarcely studied. The aim of this study is to investigate the effect of parenting styles on MDD in Chinese freshmen.

**Methods:**

A total of 9,928 Chinese freshmen were recruited in 2018. 6985 valid questionnaires were collected at one-year follow-up. Composite International Diagnostic Interview 3.0 (CIDI-3.0) was used for the diagnosis of MDD. Egna Minnen Beträffande Uppfostran (EMBU) questionnaire and Beck Depression Inventory-II (BDI-II) were used to assess parenting styles and baseline depressive symptoms, respectively. The associations between parenting styles and MDD incidence was analyzed with logistic regression.

**Results:**

The incidence of MDD in freshmen was 2.23% (95%CI: 1.91-2.60%). Maternal overprotection (OR = 1.03, 95%CI: 1.01–1.05) and disharmony relationship between parents (OR = 2.35, 95% CI: 1.42–3.89) increased the risk of new-onset MDD in freshmen, respectively. Mild depressive symptoms (OR = 2.06, 95%CI: 1.06–4.02), moderate (OR = 4.64, 95%CI: 2.55–8.44) and severe depressive symptoms (OR = 7.46, 95%CI: 2.71–20.52) at baseline increased the risk of new-onset MDD.

**Conclusions:**

Maternal overprotection, disharmony relationship between parents and baseline depressive symptoms are risk factors for new-onset MDD in Chinese freshmen.

**Supplementary Information:**

The online version contains supplementary material available at 10.1186/s12888-023-04712-0.

## Background

Major depressive disorder (MDD) is one of the leading causes of disability worldwide and one of the most common mental disorders in college students [1]. MDD causes difficulties in daily function by decreasing the ability to maintain a job, perform daily activities or social functions, and is also associated with a significant risk of suicide [2]. Therefore, it is necessary to understand potential risk factors for MDD in college students to formulate appropriate measures for prevention and early interventions.

MDD is diagnosed when either depressed mood or a lack of pleasure in usual activities is present continuously for 2 weeks or more and is accompanied by decreased energy, anhedonia, insomnia or hypersomnia, and psychomotor agitation or retardation [3, 4]. Among numerous factors predicting the onset of MDD, baseline depressive symptoms is one of them [5, 6]. Sociodemographic variables including gender [7], only child [8], the marital status of parents [9–11] and family atmosphere [12] play a significant role in the formation of depressive disorder in college students. The majority of studies show that females are more likely to suffer from depression than males [7]. Moreover, only child positively associated with depressive disorder [8]. Depression and other psychological problems are more common in individuals from divorced families [10, 11]. In addition, prior research has demonstrated that more frequent family conflicts and family disharmony are significantly related to depression [12, 13].

Parenting styles refer to the general attitudes and behaviors of parents raising and educating their children [14]. Parenting styles are important social factors in the formation of personal character and attitude, affecting a person’s life, far beyond the boundaries of childhood and adolescence [15]. Parenting styles are essential and adjustable factors contributing to the mental health of adolescents [16, 17]. For example, negative parenting styles can reduce the psychological resilience and stress ability of adolescents [18]. Individuals with authoritative parenting styles are less likely to suffer from depression and have better mental health, while authoritarian parenting styles are positively associated with students’ depression [19–21]. In addition, both paternal and maternal rejection, overprotection and punishment are positively correlated with depressive symptoms but not MDD in college students, while paternal and maternal warmth is negatively correlated with depressive symptoms [12, 22].

The association between parenting styles and the incidence of MDD is less investigated although a large number of studies have shown that parenting styles is positively associated with the incidence of depressive symptoms. Moreover, the causal role of parenting styles in MDD is scarcely studied as most studies are cross-sectional studies and focusing on depressive symptoms but not MDD. In addition, freshmen are known to encounter an increased level of stress and face challenging circumstances and high expectations [23]. Furthermore, parenting styles in China might be different from that in Western countries, e.g., Chinese parents are more likely to endorse an authoritarian parenting style [24, 25]. Therefore, we aim to determine the causal role of perceived parenting styles in MDD in Chinese freshmen with a longitudinal approach.

## Methods

### Participants and procedures of data collection

A total of 9,928 Chinese freshmen from two medical universities located in three cities (Jining, Weifang, Rizhao) in Shandong Province, P.R. China. were recruited in this cohort study by using cluster sampling method. All freshmen of grades 2017 and 2018 in two universities were recruited, 8079 (81.4%) among them provided baseline data. 7,550 (93.5%) of them completed one-year follow-up survey in 2019 (**Supplementary Fig. 1**). Shandong province is located in the north - central part of China, and is at the upper-middle level in terms of economic development and socioeconomic status, compared to other provinces in China. The majors of the two universities cover medicine and pharmacy, science and engineering, liberal arts etc. In addition, freshmen in the two universities came from 24 different provinces including large and middle urban cities, rural counties, and villages.

The data collection was carried out by using a total of 365 computers in the libraries of the three campuses, all of which were equipped with computer-assisted self-administration system. The logical checks and jumps were embedded in the survey system. Participants were divided into groups and assigned specific time to complete the survey according to the class. Before data collection, one day training for the investigators including research background, data collection procedures, the use of CIDI-3.0, potential questions and standardized answers was provided by the research team and the developers of Chinese Composite International Diagnostic Interview (CIDI) [26]. Investigator used standardized instruction and each of them was responsible for a class with 40–60 students at a time. All recruited participants completed the questionnaire independently. To ensure the confidentiality of participants and the reliability of the results, the questionnaires were anonymous. And all participants must submit their unique ID numbers generated at baseline survey. In order to ensure the quality of the questionnaires, the physical address of each smartphone or computer was accepted only once. When the participants answered the questionnaires and uploaded it many times, the physical address will be rejected. After the participants have completed the survey and submitted all the answers, the data will be uploaded directly to the local server at Jining Medical University. Participation was completely voluntary, the participants or their guardian (if younger than 18) signed an informed consent form before the interview. The research scheme was approved by the Medical Ethics Committee of Jining Medical University.

### Measures

#### Major depressive disorder

Version 3.0 of the Composite International Diagnostic Interview (CIDI-3.0) was initially developed for use in the World Health Organization-World Mental Health (WHO-WMH) survey and optimized for better effectiveness in community surveys [27]. CIDI-3.0 was used to assess lifetime and 12-month occurrence of DSM-IV disorders including MDD, dysthymia, generalized anxiety disorder, another anxiety disorder and/or alcohol abuse or dependence [28, 29]. Prior literature has found that (1) the sensitivity of CIDI-3.0 screening was between 60.4% and 93.1%, the specificity was between 33.6% and 92.7%, the positive predictive value was between 60.1% and 95.1%, and the negative predictive value was between 68.1% and 93.7%; (2) the specificity of CIDI-3.0 diagnosis was between 97.1% and 98.9%, the sensitivity was between 33.3% and 70.3%, the positive predictive value was between 66.7% and 95.7%, and the negative predictive value was between 87.7% and 95.4%; (3) the diagnostic consistency of investigators Kappa = 0.78; (4) the test-retest reliability Kappa was between 0.737 and 1.0 [26]. The test-retest reliability of the questionnaire is satisfactory, and it is effective in screening major depressive disorders and psychotic disorders. It can be used as a screening and diagnostic tool for epidemiological investigation of mental disorders in China, and can be used by non-psychiatric professionals and investigators, which is more in line with Chinese cultural background and language habits [26]. In this study, 8079 freshmen completed the CIDI-3.0 at baseline to evaluate lifetime MDD at baseline. Among them, 7,550 participants completed CIDI-3.0 at one-year follow-up survey. 407 participants diagnosed with MDD at baseline, and 158 participants with self-reported bipolar disorder were excluded from the 7,550 follow-up participants. Thus, we evaluated the new-onset MDD during the one-year follow-up in the remaining 6985 participants.

#### Perceived parenting styles

Perceived parenting styles was measured by using Egna Minnen Beträffande Uppfostran (EMBU). This questionnaire was designed for children to evaluate their parenting styles compiled by Perris, Department of Psychiatry, Umea University, Sweden, and later revised by Yue, China Medical University (1993) [30, 31]. The revised Chinese version includes 66 items and 11 dimensions, each item is scored on a 4-point scale (1 = never, 2 = occasionally, 3 = Frequently, and 4 = always). Items are combined within each dimension to create composite scores. Father’s parenting styles include 58 items and 6 dimensions: emotional warmth (19–76 points), punishment (12–48 points), favoring subject (5–20 points), rejection (6–24 points), overprotection (6–24 points), and control attempts (10–40 points). And mother’s parenting styles have 57 items and 5 dimensions: emotional warmth (19–76 points), punishment (9–36 points), favoring subject (5–20 points), rejection (8–32 points), overprotection (16–64 points). Higher scores demonstrating higher levels of parental behaviors. The homogeneity reliability coefficient of all factors ranged from 0.46 to 0.88, with an average of 0.75. The split-half reliability ranged from 0.5 to 0.91, with an average of 0.76. The test-retest reliability ranged from 0.67 to 0.89, with an average of 0.776 [31]. Since, Yue did a lot of research in China based on the EMBU scale and adapted the EMBU scale to the actual situation in China, many Chinese scholars have also adopted their modified scales, and all of these studies have confirmed that their scales have good reliability and validity [32]. Though favoritism has been identified to be culture-specific, cross-cultural stability of the EMBU based on data from Chinese subjects is satisfactory [33, 34]. Cronbach’s alpha for the EMBU in the current study was 0.94.

#### Baseline depressive symptoms

Beck Depression Inventory-II, a self-rating scale, was used to evaluate the severity of depressive symptoms within the past two weeks [35]. It consists of 21 items, each with four points between 0 and 3, corresponding to four different degrees of the same event, with higher scores indicating more severe depressive symptoms. A total score of 0–13 indicates no depressive symptoms, 14–19 mild, 20–28 moderate, and 29–63 severe. Chinese Version of the BDI-II (BDI-II-C) has good reliability and validity with Cronbach alpha of 0.94, which is similar to the English version [36]. The application was approved by authors [36]. In Chinese freshmen, the Cronbach alpha was 0.85 [37]. The Cronbach alpha in the current study was 0.91. In this study, BDI-II was used to evaluate baseline depressive symptoms.

#### Socio-demographic characteristics

Socio-demographic characteristics of freshmen were obtained from self-prepared general information questionnaire, including gender, residence, only-child, major, the marital status of parents (measured by the question “What is the marital status of your parents”, with 4 answers: “Married”, “Divorced”, “Widowed” and “Separated”), parent’s relationship (measured by the question: “How would you rate your parents’ husband and wife relationship”, the response options were “Not in harmony”, “General” and “Harmonious”), marriage satisfaction (measured by the question: “Subjectively, how satisfied are you with your parents’ marital status”, with a 10-point rating scale, where 1 = very dissatisfied and 10 = very satisfied) and overall feeling about family atmosphere (measured by the question: “So far, what is your overall feeling about the family atmosphere”, with 4 response options: “1. Fine 2. Neutrally 3. Badly 4. Not sure”).

### Statistical analyses

SPSS 28.0 Version was used for all statistical analysis. T-test and Chi-square test (χ^2^) were applied for statistical analysis. Pearson’s chi-square test (theoretical number (T) ≥ 5 and sample size n ≥ 40) and likelihood-ratio Chi-square test (1 ≤ T < 5, n ≥ 40) were used, respectively. Bivariate correlation matrix using Spearman rank correlation analysis and Pearson correlation analysis was performed to determine the simple correlation and multicollinearity between different socio-demographic characteristics and parenting styles, and among dimensions of parenting styles. Forward and Backward methods based on conditional parameter estimation, partial maximum likelihood estimation, Wald statistics and Enter method were used to generate prediction models and avoid the influence of multicollinearity. Linear regression analysis was used to explore the predictive effect of parenting styles on baseline depressive symptoms score. Uni-variable logistic regression and multivariable logistic regression with Forward method based on partial maximum likelihood estimation (Forward:LR) were used to identify predictors of new-onset MDD. *P <* 0.05 was considered statistically significant. The selection of independent variables for multivariable logistic regression was based on the results of prediction analysis, professional knowledge and clinical practice. A total of 11 variables were included in the multivariable logistic regression analysis, which were gender, residence, major, only-child, marital status of parents, parents’ relationship, parents’ marriage satisfaction, overall feeling about family atmosphere, baseline depressive symptoms, father’s parenting style and mother’s parenting style.

## Results

### The baseline socio-demographic characteristics of follow-up completers and non-completers

8079 and 7550 participants completed the baseline survey and one-year follow-up survey respectively. 529 participants (6.5%) are lost to follow-up. The mean age of follow-up completers and non-completers are 18.36 ± 0.87 and 18.35 ± 0.79 (t= -0.40; P > 0.05), respectively. As shown in Table 1, gender (χ^2^ = 5.20; *P* = 0.023), major (χ^2^ = 254.87; *P* < 0.001) and the marital status of parents (χ^2^ = 7.52; *P* = 0.006) were significantly different between follow-up completers and non-completers.

**Table 1:** The baseline socio-demographic characteristics of follow-up completers and non-completers

Variables		Categories	Completers (%)	Non-completers (%)	χ^2^	*P*
Gender						
		Males	2989(92.7)	236(7.3)	5.20	0.023
		Females	4561(94.0)	293(6.0)		
Residence						
		Urban	2793(93.3)	202(6.7)	0.30	0.583
		Rural	4757(93.6)	327(6.4)		
Major						
		Medicine	5516(96.3)	214(3.7)	254.87	< 0.001
		Non-medicine	2034(86.6)	315(13.4)		
Only-child						
		No	4593(93.3)	329(6.7)	0.80	0.372
		Yes	2885(93.8)	190(6.2)		
The marital status of parents						
		In marriage	7114(93.6)	483(6.4)	7.52	0.006
		Others	436(90.5)	46(9.5)		
Parents’ relationship						
		Not in harmony	476(93.7)	32(6.3)		
		General	2178(93.6)	150(6.4)	0.04	0.980
		Harmonious	4826(93.5)	336(6.5)		
Parents’ marriage satisfaction level (scores)						
		P25 (1–6)	1558(92.7)	122(7.3)		
		P50 (7–8)	2090(93.8)	139(6.2)	5.12	0.163
		P75 (9)	1461(94.6)	84(5.4)		
		P100 (10)	2371(93.2)	173(6.8)		
Overall feeling about family atmosphere						
		Fine	5736(93.8)	378(6.2)		
		Neutrally	1388(92.7)	109(7.3)	4.28	0.233
		Badly	249(91.5)	23(8.5)		
		Not sure	107(93.0)	8(7.0)		
Baseline depressive symptoms						
		No (0–13)	7008(93.6)	483(6.4)		
		Mild (14–19)	257(91.8)	23(8.2)	3.06	0.383
		Moderate (20–28)	173(95.6)	8(4.4)		
		Severe (29–63)	42(91.3)	4(8.7)		

### Socio-demographic characteristics of follow-up participants with new-onset MDD and Non-MDD

158 participants with self-reported bipolar disorder and schizophrenia and 407 cases diagnosed as MDD with CIDI 3.0 at baseline were excluded. 6985 of 7550 collected questionnaires were valid at 1-year follow-up. The mean age of New-onset MDD and Non-MDD participants are 18.29 ± 0.89 and 18.37 ± 0.86 (t = 1.09; P > 0.05), respectively. Among 6985 participants, 158 were diagnosed with New-onset MDD (Table 2). The missing values of Parents’ relationship, Parents’ marriage satisfaction level and Overall feeling about family atmosphere were 1 (0.63%) in the New-onset MDD group and 45 (0.66%) in the Non-MDD group. Moreover, the missing values in the New-onset MDD group and the Non-MDD group of baseline depressive symptoms were 2 (1.27%) and 44 (0.64%), respectively. The percentage of the above missing values were less than 2%, which can be considered inconsequential with reference to the general standard of 5% [38]. There was no significant difference in gender (χ^2^ = 1.41; *P* > 0.05), residence (χ^2^ = 0.04; *P* > 0.05), major (χ^2^ = 0.04; P > 0.05), only-child status (χ^2^ = 0.00; *P* > 0.05), marital status of parents (χ^2^ = 0.01; *P* > 0.05) between new-onset MDD group and non-MDD group. However, significant differences were revealed among the remaining variables, including parents’ relationship (χ^2^ = 21.54; *P* < 0.001), parents’ marriage satisfaction level (χ^2^ = 16.14; P = 0.001), overall feeling about family atmosphere (χ^2^ = 19.32; *P* < 0.001) and baseline depressive symptoms (χ^2^ = 42.36; *P* < 0.001).

**Table 2:** Socio-demographic characteristics of New-onset MDD and Non-MDD participants

Variables	Categories	New-onset MDD (%)	Non-MDD (%)	χ^2^	*P*
Gender					
	Males	55(2.0)	2695(98.0)	1.41	0.235
	Females	103(2.4)	4132(97.6)		
Residence					
	Urban	59(2.3)	2495(97.7)	0.04	0.837
	Rural	99(2.2)	4332(97.8)		
Major					
	Medicine	115(2.2)	5018(97.8)	0.04	0.840
	Non-medicine	43(2.3)	1809(97.7)		
Only-child					
	No	97(2.3)	4161(97.7)	0.00	0.969
	Yes	61(2.3)	2600(97.7)		
The marital status of parents					
	In marriage	149(2.3)	6447(97.7)	0.01	0.944
	Others	9(2.3)	380(97.7)		
Parents’ relationship					
	Not in harmony	21(5.0)	397(95.0)		
	General	55(2.8)	1923(97.2)	21.54	< 0.001
	Harmonious	81(1.8)	4462(98.2)		
Parents’ marriage satisfaction level (scores)					
	P25 (1–6)	46(3.3)	1346(96.7)		
	P50 (7–8)	52(2.7)	1873(97.3)	16.14	0.001
	P75 (9)	27(2.0)	1357(98.0)		
	P100 (10)	32(1.4)	2206(98.6)		
Overall feeling about family atmosphere					
	Fine	102(1.9)	5292(98.1)		
	Neutrally	39(3.2)	1195(96.8)	19.32	< 0.001
	Badly	14(6.5)	201(93.5)		
	Not sure	2(2.1)	94(97.9)		
Baseline depressive symptoms					
	No (0–13)	127(1.9)	6440(98.1)		
	Mild (14–19)	10(4.7)	201(95.3)	42.36	< 0.001
	Moderate (20–28)	14(10.6)	118(89.4)		
	Severe (29–63)	5(17.2)	24(82.8)		

### Perceived parenting styles of follow-up participants with new-onset MDD and Non-MDD

158 new-onset MDD cases were diagnosed at 1-year follow-up, accounting for 2.23% (95%CI: 1.91-2.60%) of freshmen who had completed the baseline and 1-year follow-up survey [39]. The missing values in the New-onset MDD group and the Non-MDD group were 1 (0.63%) and 45 (0.66%), respectively. It results in 157 in new-onset MDD group and 6782 in Non-MDD group (Table 3). No difference was detected in dimensions of parental favoring and paternal control attempt in participants with new-onset MDD and non-MDD (*P* > 0.05). However, significant differences were revealed in the remaining eight of the eleven dimensions of parental rearing style (*P* < 0.05). The scores of paternal emotional warmth and maternal emotional warmth were significantly lower in new-onset MDD group than the scores in non-MDD group, 50.84 ± 10.59 vs. 54.23 ± 10.73 and 51.98 ± 10.78 vs. 54.73 ± 10.45, respectively (Table 3). In addition, the scores of paternal punishment (17.94 ± 5.17 vs. 16.69 ± 5.00), maternal punishment (13.34 ± 4.40 vs. 11.98 ± 3.77), paternal rejection (9.43 ± 2.87 vs. 8.85 ± 2.73), maternal rejection (12.97 ± 3.81 vs. 12.06 ± 3.69), paternal overprotection (12.61 ± 2.56 vs. 11.97 ± 2.81) and maternal overprotection (35.00 ± 7.41 vs. 32.71 ± 6.58) were significantly higher in new-onset MDD group than the scores in non-MDD group (Table 3). These results suggest that parental emotional warmth is a protective factor while parental punishment, rejection and overprotection are risk factors for new-onset MDD.

**Table 3:** EMBU scores of New-onset MDD and Non-MDD participants

Domains	Dimensional variables	New-onset MDD (M ± SD) (n_1_ = 157)	Non-MDD (M ± SD) (n_2_ = 6782)	t	*P*
Paternal					
	Emotional warmth	50.84 ± 10.59	54.23 ± 10.73	3.91	< 0.001
	Punishment	17.94 ± 5.17	16.69 ± 5.00	-3.09	0.002
	Control attempt	19.90 ± 4.64	19.32 ± 3.99	-1.81	0.071
	Favoring	10.17 ± 3.64	9.91 ± 3.60	-0.89	0.373
	Rejection	9.43 ± 2.87	8.85 ± 2.73	-2.62	0.009
	Overprotection	12.61 ± 2.56	11.97 ± 2.81	-2.83	0.005
Maternal					
	Emotional warmth	51.98 ± 10.78	54.73 ± 10.45	3.26	0.001
	Punishment	13.34 ± 4.40	11.98 ± 3.77	-3.86	< 0.001
	Favoring	10.11 ± 3.62	9.99 ± 3.60	-0.42	0.677
	Rejection	12.97 ± 3.81	12.06 ± 3.69	-3.04	0.002
	Overprotection	35.00 ± 7.41	32.71 ± 6.58	-4.31	< 0.001

### Regression analysis of perceived parenting styles and risk of new-onset MDD

Univariate regression analysis was further performed. Parents’ relationship, parents’ marriage satisfaction level and the overall feeling about family atmosphere were included in the univariate regression prediction model (Table 4). Eight dimensions of parenting styles had predictive effects on new-onset MDD, including paternal emotional warmth (OR = 0.97, 95%CI: 0.96–0.99), maternal emotional warmth (OR = 0.98, 95%CI: 0.96–0.99), paternal punishment (OR = 1.04, 95%CI: 1.02–1.07), maternal punishment (OR = 1.08, 95%CI: 1.04–1.11), paternal rejection (OR = 1.07, 95%CI: 1.02–1.13), maternal rejection (OR = 1.06, 95%CI: 1.02–1.10), paternal overprotection (OR = 1.08, 95%CI: 1.02–1.14) and maternal overprotection (OR = 1.05, 95%CI: 1.03–1.07) (Table 4). However, the dimensions of paternal favoring (OR = 1.02, 95%CI: 0.98–1.07), paternal control attempt (OR = 1.04, 95%CI: 1.00-1.08) and maternal favoring (OR = 1.01, 95%CI: 0.97–1.05) had no predictive effect on new-onset MDD. In addition, mild depressive symptoms (OR = 2.52, 95%CI: 1.31–4.88), moderate depressive symptoms (OR = 6.02, 95%CI: 3.36–10.76) and severe depressive symptoms (OR = 10.56, 95%CI: 3.97–28.13) at baseline were predictors of new-onset MDD (Table 4).

**Table 4:** Univariate logistic regression of predictive factors of New-onset MDD

Variables	Categories	B (SE)	Wald χ2	*P*	OR	95% CI	
						Lower	Upper
Gender	Males/Females	-0.20(0.17)	1.40	0.236	0.82	0.59	1.14
Residence	Urban/Rural	0.03(0.17)	0.04	0.837	1.04	0.75	1.43
Major	Non-medicine/medicine	0.04(0.18)	0.04	0.840	1.04	0.73	1.48
Only-child	No/Yes	-0.01(0.17)	0.00	0.969	0.99	0.72	1.37
Marital status of parents	In marriage/Others	-0.02(0.35)	0.01	0.944	0.98	0.49	1.93
Parents’ relationship							
	Harmonious	reference					
	General	0.46(0.18)	6.61	0.010	1.58	1.11	2.23
	Not in harmony	1.07(0.25)	18.24	< 0.001	2.91	1.78	4.76
Parents’ marriage satisfaction level (scores)							
	P100 (10)	reference					
	P25 (1–6)	0.86(0.23)	13.55	< 0.001	2.36	1.49	3.72
	P50 (7–8)	0.65(0.23)	8.19	0.004	1.91	1.23	2.99
	P75 (9)	0.32(0.26)	1.44	0.231	1.37	0.82	2.30
Overall feeling about family atmosphere							
	Fine	reference					
	Neutrally	0.53(0.19)	7.61	0.006	1.69	1.17	2.46
	Badly	1.29(0.29)	19.10	< 0.001	3.61	2.03	6.43
	Not sure	0.10(0.72)	0.02	0.891	1.10	0.27	4.54
Baseline depressive symptoms							
	No (0–13)	reference					
	Mild (14–19)	0.93(0.34)	7.58	0.006	2.52	1.31	4.88
	Moderate (20–28)	1.79(0.30)	36.62	< 0.001	6.02	3.36	10.76
	Severe (29–63)	2.36(0.50)	22.26	< 0.001	10.56	3.97	28.13
Father’s parenting style							
	Emotional warmth	-0.03(0.01)	15.17	< 0.001	0.97	0.96	0.99
	Punishment	0.04(0.01)	9.48	0.002	1.04	1.02	1.07
	Control attempt	0.04(0.02)	3.26	0.071	1.04	1.00	1.08
	Favoring	0.02(0.02)	0.79	0.373	1.02	0.98	1.07
	Rejection	0.07(0.03)	6.84	0.009	1.07	1.02	1.13
	Overprotection	0.08(0.03)	7.99	0.005	1.08	1.02	1.14
Mother’s parenting style							
	Emotional warmth	-0.02(0.01)	10.55	0.001	0.98	0.96	0.99
	Punishment	0.07(0.02)	19.52	< 0.001	1.08	1.04	1.11
	Favoring	0.01(0.02)	0.17	0.677	1.01	0.97	1.05
	Rejection	0.06(0.02)	9.19	0.002	1.06	1.02	1.10
	Overprotection	0.05(0.01)	18.51	< 0.001	1.05	1.03	1.07

To test the correlation and potential multicollinearity between the independent variables shown in Table 4, Bivariate correlation matrix using Spearman rank correlation analysis was performed to determine the simple correlation and multicollinearity between parenting styles and 9 socio-demographic characteristic variables including gender, residence, major, only child, the marital status of parents, parental relationship, parents’ marriage satisfaction level, overall feeling about family atmosphere and baseline depressive symptoms (Supplementary Table 1). Bivariate correlation matrix using Pearson correlation analysis was performed to determine the simple correlation and multicollinearity between different dimensions of parenting styles (Supplementary Table 2). We found that most correlation coefficients between socio-demographic characteristics and parenting styles were less than 0.50 (Supplementary Table 1). The significant correlations with low coefficients indicated that there was no existence of multicollinearity among variables for predicting new-onset MDD [40]. However, there were high positive correlation and very high positive correlation (correlation coefficients > 0.70 and > 0.90) [40] among several dimensions of parenting styles in EMBU, such as paternal and maternal emotional warmth, paternal punishment and rejection, paternal punishment and maternal favoring etc. (Supplementary Table 2), indicating the potential multicollinearity may exist. Forward regression, backward regression and stepwise regression is one of the common methods to solve multicollinearity [41]. In the current study, combining the results of prediction analysis, professional knowledge and clinical practice, the best prediction model was selected among various prediction models to identify predictors of new-onset MDD, which was obtained by using multivariable logistic regression with Forward method based on partial maximum likelihood estimation (Forward : LR), Wald statistics and Enter method (Table 5). The disharmonious parental relationship significantly increased the risk of new-onset MDD (OR = 2.35, 95%CI: 1.42–3.89). Maternal overprotection (OR = 1.03, 95%CI: 1.01–1.05) was a risk factor for new-onset MDD. Moreover, the mild depressive symptoms (OR = 2.06, 95%CI: 1.06–4.02), moderate (OR = 4.64, 95%CI: 2.55–8.44) and severe depressive symptoms (OR = 7.46, 95%CI: 2.71–20.52) strongly increased the risk of new-onset MDD.

**Table 5:** Multivariate logistic regression of predictive factors of New-onset MDD

Variables	B (SE)	Wald χ^2^	*P*	OR	95% CI	
					Lower	Upper
Parents’ relationship						
Harmonious	reference					
General	0.31 (0.18)	2.84	0.092	1.36	0.95	1.93
Not in harmony	0.85 (0.26)	11.00	0.001	2.35	1.42	3.89
Baseline depressive symptoms						
No (0–13)	reference					
Mild (14–19)	0.72 (0.34)	4.51	0.034	2.06	1.06	4.02
Moderate (20–28)	1.53 (0.31)	25.15	< 0.001	4.64	2.55	8.44
Severe (29–63)	2.01(0.52)	15.13	< 0.001	7.46	2.71	20.52
Maternal overprotection	0.03(0.01)	6.22	0.013	1.03	1.01	1.05

## Discussion

### Main findings

In this one-year longitudinal study, incidence of MDD and predictive effect of perceived parenting styles on new-onset MDD in a representative sample of college freshmen were estimated. The 1-year incidence of MDD in freshmen was 2.23% (95%CI: 1.91-2.60%). Maternal overprotection and disharmony relationship between parents are risk factors for the new-onset MDD in freshmen.

In a cohort study of freshmen from Leuven College Surveys, the incidence rate of MDD was 6.9% [1], which was much higher than that reported in this study. However, previous Chinese studies have reported that the incidence of MDD among college students was 2.4-4.0% [42, 43]. Similarly, the prevalence of MDD in China is lower than that in North America and Western Europe, while similar to that in other Asian countries [42]. Geographical/ethnic/racial differences may be one of the reasons for these gaps. Moreover, Chinese people are reluctant to report psychological symptoms in face-to-face interviews because of stigma [13]. In our study, we found that gender has no significant effect on MDD in Chinese freshmen. This may be related to the different trajectories of boys and girls depression levels in adolescence. Hou & Chen found that the depression of both boys and girls increased linearly during the age of 10 ~ 19, but the results of model fitting showed that the growth trend of girls’ depression extended to adulthood, and then declined, showing an inverted U-shaped development curve [44]. Moreover, although western studies have shown that the depression of girls is significantly higher than that of boys [45], Chinese studies have found that the depression of adolescent boys is as high as that of girls [46], and even shows that the depression of boys is higher than that of girls [47].

Maternal overprotection increase risk of new-onset MDD in Chinese freshmen, which are highly supported by previous studies. First, parental overprotection is a strong predictor for depression in Chinese college students [48, 49]. Moreover, parental overprotection leads to lower life satisfaction, lower self-efficacy and higher levels of depression [22, 50]. Parental overprotection refers to parental behaviours reflecting control, overprotection, intrusion, excessive contact and prevention of independent behaviour as opposed to allowance of independence and autonomy [51]. As a result of the long-term overprotection of parents, their offspring have no independent opinions facing problems or difficulties and have limited ability to cope with problems or difficulties, causing psychological problems more easily. The negative effects of this overprotection may not be apparent in childhood and adolescence when living with parents. However, the negative effects may show up when they left home, e.g. college students [48].

Positive parenting styles such as paternal emotional warmth was negatively correlated with the baseline depressive symptoms, while negative parenting style such as paternal punishment, maternal overprotection was positively correlated with the baseline depressive symptom score. Moreover, baseline depressive symptoms increased the risk of new-onset MDD at one-year follow-up in freshmen which is consistent with the previous study that subclinical depressive symptoms predict later onset of a full major depressive episode [52]. It suggests that the screening and intervention of depressive symptoms in freshmen may be an effective approach to reduce MDD incidence.

Disharmony relationship between parents increase risk of new-onset MDD. This may be related to the domestic violence and conflict. Studies have shown that children’s exposure to parental violence increases negative emotions, including pain, fear, anger and worry, as well as increased emotional sensitivity to parental conflict. It makes the offspring more likely to suffer from mental disorders [53]. College students experiencing parental violence were prone to have anxiety symptoms, depressive symptoms, and the comorbidity of anxiety and depressive symptoms in a sample of Chinese students [8]. However, the neurobiological mechanism of how parental disharmony affecting the mental health of offspring is still unclear, which needs to be further explored in the future.

### Limitations

There were several limitations in this study. Although this is a cohort study, the questionnaire survey via self-report may have caused recall bias. Secondly, freshmen rather than their parents had completed the EMBU questionnaire survey, which might amplify the relationship between parenting styles and new-onset MDD due to their subjective feelings. Thirdly, bias due to the loss of follow-up may exist. Finally, family history, the life/traumatic events, interpersonal stress etc. was not included in this study.

## Conclusions

Our findings revealed that maternal overprotection and disharmony relationship between parents are the risk factors for new-onset MDD in Chinese freshmen, suggesting intervention and adjustment of parenting styles may be beneficial for the prevention of MDD in college students. Thus, parenting programs promoting positive parenting styles should be encouraged.

## Electronic supplementary material

Below is the link to the electronic supplementary material.

Supplementary Material

## Data Availability

Original data that support the findings of this study are available on request from Dr. Yan Liu (email: hakunaly@163.com).

## Abbreviations

MDD: Major depressive disorder
CIDI-3.0: Version 3.0 of the Composite International Diagnostic Interview
WHO-WMH: World Health Organization-World Mental Health
EMBU: Egna Minnen Beträffande Uppfostran
BDI-II: Beck Depression Inventory-II
BDI-II-C: Chinese Version of the BDI-II
Forward: LR: Forward method based on partial maximum likelihood estimation
