# Supplementary material for: Perceived parenting styles and incidence of major depressive disorder: results from a 6985 freshmen cohort study
Source: BMC Psychiatry. 2023 Apr 5;23:230. doi: 10.1186/s12888-023-04712-0 (PMC10074813; doi:10.1186/s12888-023-04712-0)
Supplement: Supplementary file 1 — Supplementary Material [file 12888_2023_4712_MOESM1_ESM.docx]

**Supplementary Figure 1. Flow-chart of sampling**

| 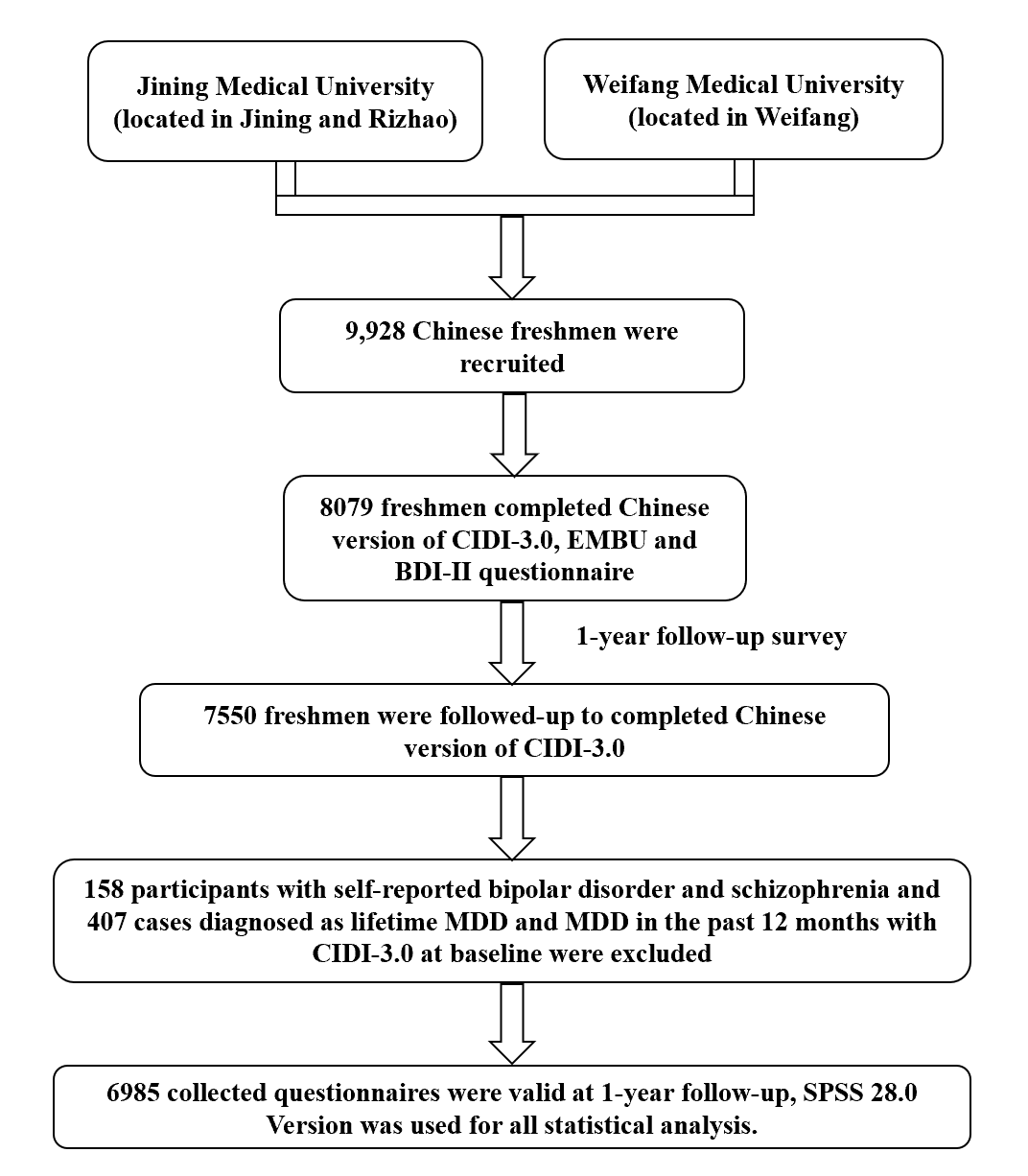 |
| --- |

**Supplementary Table 1**

**Spearman correlation coefficients between socio-demographic characteristics and perceived parenting styles**

| Variables | 1 | 2 | 3 | 4 | 5 | 6 | 7 | 8 | 9 |
| --- | --- | --- | --- | --- | --- | --- | --- | --- | --- |
| 1 | 1 |  |  |  |  |  |  |  |  |
| 2 | 0.07* | 1 |  |  |  |  |  |  |  |
| 3 | -0.27* | -0.45* | 1 |  |  |  |  |  |  |
| 4 | -0.05* | -0.07* | 0.05* | 1 |  |  |  |  |  |
| 5 | 0.01 | -0.01 | -0.01 | -0.02# | 1 |  |  |  |  |
| 6 | -0.05 | 0.01 | 0.00 | 0.01 | -0.57* | 1 |  |  |  |
| 7 | 0.01 | -0.05* | 0.06* | 0.01 | 0.20* | -0.25* | 1 |  |  |
| 8 | -0.01 | -0.02 | 0.01 | -0.01 | 0.15* | -0.11* | 0.05* | 1 |  |
| 9 | -0.08* | 0.01 | 0.01 | 0.03* | -0.55* | 0.67* | -0.20* | -0.12* | 1 |
| PE | 0.04* | -0.09* | 0.10* | 0.04* | -0.39* | 0.35* | -0.11* | -0.15* | 0.40* |
| PP | -0.18* | -0.01 | 0.02 | -0.02 | 0.24* | -0.17* | 0.00 | 0.17* | -0.22* |
| PC | -0.15* | -0.06* | 0.07* | 0.00 | 0.12* | -0.07* | -0.03* | 0.12* | -0.11* |
| PF | -0.01 | -0.10* | 0.16* | 0.06* | -0.02 | 0.00 | -0.01 | 0.04* | 0.00 |
| PR | -0.16* | -0.01 | 0.03* | -0.02 | 0.24* | -0.19* | 0.01 | 0.17* | -0.25* |
| PO | -0.11* | 0.00 | 0.02# | 0.00 | 0.06* | -0.05* | -0.04* | 0.13* | -0.06* |
| ME | 0.05* | -0.12* | 0.13* | 0.04* | -0.33* | 0.27* | -0.07* | -0.14* | 0.34* |
| MO | -0.13* | -0.08* | 0.10* | 0.02 | 0.15* | -0.13* | -0.00 | 0.16* | -0.16* |
| MR | -0.11* | -0.01 | 0.01 | -0.02 | 0.25* | -0.21* | 0.02* | 0.18* | -0.26* |
| MP | -0.14* | -0.02# | 0.02 | -0.03* | 0.25* | -0.20* | 0.04* | 0.18* | -0.24* |
| MF | -0.02 | -0.12* | 0.17* | 0.06* | 0.01 | -0.03# | 0.02# | 0.05* | -0.02# |

a #, *p<0.05* (double tail), the correlation is significant; *, *p<0.01* (double tail), the correlation is significant;

b 1:Gender; 2:Residence; 3:Only-child; 4:Major; 5:Overall feeling about family atmosphere; 6:Parents’ relationship; 7:The marital status of parents; 8:Baseline depressive symptoms; 9:Parents’ marriage satisfaction level (scores); PE: Paternal Emotional warmth; PP: Paternal Punishment; PC: Paternal Control attempt; PF: Paternal Favoring; PR: Paternal Rejection; PO: Paternal Overprotection; ME: Maternal Emotional warmth; MO: Maternal Overprotection; MR: Maternal Rejection; MP: Maternal Punishment; MF: Maternal Favoring.

**Supplementary Table 2**

**Pearson correlation coefficients between perceived parenting styles**

| Variables | PE | PP | PC | PF | PR | PO | ME | MO | MR | MP | MF |
| --- | --- | --- | --- | --- | --- | --- | --- | --- | --- | --- | --- |
| PE | 1 |  |  |  |  |  |  |  |  |  |  |
| PP | -0.37* | 1 |  |  |  |  |  |  |  |  |  |
| PC | -0.17* | 0.61* | 1 |  |  |  |  |  |  |  |  |
| PF | 0.23* | 0.14* | 0.14* | 1 |  |  |  |  |  |  |  |
| PR | -0.34* | **0.78*** | 0.60* | 0.15* | 1 |  |  |  |  |  |  |
| PO | 0.07 | 0.47* | 0.60* | 0.23* | 0.51* | 1 |  |  |  |  |  |
| ME | **0.88*** | -0.32* | -0.17* | 0.21* | -0.31* | -0.05 | 1 |  |  |  |  |
| MO | -0.14* | 0.50* | **0.73*** | 0.21* | 0.55* | 0.66* | -0.08* | 1 |  |  |  |
| MR | -0.34* | **0.70*** | 0.53* | 0.16* | **0.82*** | 0.50* | -0.33* | 0.63* | 1 |  |  |
| MP | -0.36* | **0.83*** | 0.49* | 0.14* | 0.69* | 0.39* | -0.37* | 0.54* | 0.77* | 1 |  |
| MF | 0.16* | 0.16* | 0.13* | **0.94*** | 0.16* | 0.21* | 0.23* | 0.23* | 0.16* | 0.15* | 1 |

a *, *p<0.01* (double tail), the correlation is significant;

b significant correlations with r > 0.70 are in bold

c PE: Paternal Emotional warmth; PP: Paternal Punishment; PC: Paternal Control attempt; PF: Paternal Favoring; PR: Paternal Rejection; PO: Paternal Overprotection; ME: Maternal Emotional warmth; MO: Maternal Overprotection; MR: Maternal Rejection; MP: Maternal Punishment; MF: Maternal Favoring.
